# Factors influencing initial choice of insulin therapy in a large international non-interventional study of people with type 2 diabetes

**DOI:** 10.1111/j.1463-1326.2012.01613.x

**Published:** 2012-10

**Authors:** N Freemantle, B Balkau, N Danchin, E Wang, M Marre, G Vespasiani, R Kawamori, P D Home

**Affiliations:** 1 Department of Primary Care and Population Health, University College LondonLondon, UK; 2 INSERM, CESP Centre for research in Epidemiology and Population Health, U1018, Epidemiology of Diabetes, Obesity and Chronic Kidney Disease Over the Lifecourse and Determinants of Early nutritionUniversity Paris Sud 11, UMRS 1018, Villejuif, France; 3 Division of Coronary Artery Disease and Intensive Cardiac CareUniversité Paris 5, Paris, France; 4 Global Medical AffairsSanofi, Bridgewater, NJ, USA; 5 Groupe Hospitalier Bichat-Claude Bernard, Assistance Publique des Hôpitaux de ParisINSERM U 695, Université Paris 7, Paris, France; 6 Diabetology and Metabolic Disorders Centre, Madonna del Soccorso HospitalSan Benedetto del Tronto, Italy; 7 Department of Medicine, Juntendo UniversityTokyo, Japan; 8 Institute of Cellular Medicine—Diabetes, Newcastle UniversityNewcastle upon Tyne, UK

**Keywords:** CREDIT study, insulin regimens, type 2 diabetes

## Abstract

**Aim:**

To use baseline characteristics of the Cardiovascular Risk Evaluation in people with type 2 Diabetes on Insulin Therapy study population to identify factors that could explain the choice of insulin therapy when beginning insulin.

**Methods:**

The source, non-interventional, longitudinal, long-term study involves 314 centres in 12 countries in five regions. People were enrolled having started any insulin regimen in the previous 12 months. To identify factors associated with the choice of insulin regimen, multivariable backward logistic regression was performed on eligible physician and participant explanatory variables.

**Results:**

Participants (N = 3031) had mean age 62 years, diabetes duration 11 years, body mass index 29.3 kg/m^2^ and an HbA1c of 9.5%. Participants in Japan had less hypertension, smoked more and used fewer concomitant medications than those of other regions. Only physician location (rural or urban) influenced the choice of insulin in Japan. In the other four-regions-combined, physician location, specialty, sex and practice type influenced choice of insulin as did participant location, baseline HbA1c, use of glucose-lowering therapies and prior insulin secretagogue use.

**Conclusion:**

Choice of initial insulin regimen was influenced by several physician and participant characteristics in Canada and Europe, but only by physician location in Japan.

## Introduction

Landmark clinical trials have established that good glycaemic control in people with type 2 diabetes mellitus (T2DM) can prevent long-term microvascular complications and may prevent macrovascular problems, associated with the condition [1–3]. Several organizations have established target glycaemic goals which, if followed, should result in improved long-term outcomes [4–6]. Because T2DM is a progressive condition, continued additions of therapies are usually needed, adding other oral glucose-lowering drugs (OGLDs) when metformin plus lifestyle measures fail to provide adequate control. When the target levels cannot be maintained by multiple OGLDs, insulin therapy is often commenced.

An expert consensus group with representatives from The American Diabetes Association and the European Association for the Study of Diabetes recommended that people with T2DM begin insulin with a basal insulin regimen [4]. However, the recommendation from the International Diabetes Federation Clinical Guidelines Task Force is broader and includes beginning with basal insulin, premix insulin or basal + mealtime insulin regimens [5]. Mealtime insulin only is another option. Any insulin regimen can be initiated with or without concomitant OGLDs [7]. While a number of both qualitative and quantitative factors have been identified as important for initiating any insulin therapy [8], it is of interest to investigate what factors are predictive for the initial choice of an insulin regimen.

The Cardiovascular Risk Evaluation in people with type 2 Diabetes on Insulin Therapy (CREDIT) study was designed to evaluate the relationship between glycaemic control and cardiovascular events in persons treated with insulin and to provide insight into current, real-world practices of the use of insulin in people with T2DM. The characteristics of people with T2DM in different countries at the time of starting insulin have been reported [9,10]. The current report uses the baseline characteristics of the CREDIT study population to identify factors that could explain the chosen strategy of insulin therapy at the time of beginning insulin.

## Methods

### Study Design and Site Selection

The CREDIT study, from which the current data are derived, is a 4-year, non-interventional, longitudinal, study involving 314 centres in 12 countries, one country in each of North America and Asia, the rest in Europe. Three of the countries, all in Eastern Europe, have emerging economies. In line with the study design, there is no fixed study visit schedule, and data are gathered in routine clinical practice, but the physicians are asked to report updated participant data on a 6-month cycle. The first participant was enrolled on 4 December 2006 and the last on 30 April 2008. The present analysis deals only with baseline data gathered at entry.

For each country, a master list was compiled of all potential participating study sites where physicians were familiar with starting insulin and regularly followed people with diabetes. The initial list was four times larger than the number of sites needed, anticipating that one site would agree to participate for every four contacted. In order to avoid cherry-picking of possibly unrepresentative diabetes care, primary care, hospital-based and diabetes centres were all included. The sites approached were then randomly selected from these lists, being careful to ensure that the study population was representative of the overall pool. A fixed ratio was maintained between primary care and specialists in France, but in Italy, nearly all people with diabetes are managed by specialists in diabetes centres. Each participating site was requested to enroll at least five and no more than 30 consecutive eligible (existing or incoming) patients visiting her/him during the recruitment period. Ethical approval according to local regulations was obtained for all study sites. Conduct of the study was in line with the standards of data collection for clinical trials, according to the Declaration of Helsinki. Written informed consent was obtained from all participants before commencement of data collation.

### Participant Selection

The primary criteria required men and women with T2DM, age >40 years, who had started insulin therapy >1 month and <6 months prior to study entry and who had an HbA1c measurement within the 3 months prior to beginning insulin. No stipulation was made as to insulin type or regimen, educational package, support staff, self-monitoring or the like.

The following definitions of baseline characteristics were used: type 2 diabetes (diabetes diagnosed at least 12 months before starting insulin and not believed to be type 1 or secondary diabetes by the investigator); smoking status (never smoked, stopped <1 year or ≥1 year, currently smokes); physical activity (walking, cycling or gardening for ≥4 h/week); history of macrovascular disease (myocardial infarction, stable angina, severe unstable angina leading to hospitalization, heart failure, stroke, transient ischaemic attack, peripheral vascular disease, myocardial revascularization, peripheral revascularization, lower limb amputation); history of microvascular disease [retinopathy, peripheral neuropathy, nephropathy including microalbuminuria (30–299 mg/24 h), macroalbuminuria (≥300 mg/24 h), renal failure (confirmed by creatinine clearance) and dialysis and/or transplantation]; hypertension (systolic blood pressure ≥130 mmHg and/or diastolic blood pressure ≥80 mmHg). Baseline data was collected retrospectively from clinical records. HbA1c values are given in National Glycohemoglobin Standardization Program units with International Federation of Clinical Chemistry values in parenthesis.

### Statistics

Analyses were performed with SAS statistical software, version 9.1 (SAS Institute, Cary, NC, USA). All data were reported and analysed using descriptive statistics. For continuous variables, 95% confidence intervals (CIs) based upon the *t*-distribution were generated.

To identify factors associated with the choice of insulin regimen at the time of beginning insulin, univariate analyses were performed on a predefined number of candidate explanatory variables (5 physician and 20 participant characteristics), with comparisons of categorical variables between subgroups made by chi-square and of continuous variables by Kruskal–Wallis tests. Multiple imputation of missing data was used for robustness purposes. All variables with <20% of missing data were kept and imputed. In all five regions, three physician and seven participant characteristics were associated with beginning insulin at p ≤ 0.20, and therefore included in both the four-regions-combined (excluding Japan) and the Japan-only multivariable analyses. Physician characteristics were geographical location (urban/rural), type of practice (office- or hospital-based or both) and age. Participant characteristics included the presence of ≥1 microvascular disease, previous diagnosis of high blood pressure, physical activity (yes/no), HbA1c, and the number of glucose-lowering therapies, biguanide use and insulin secretagogue use before beginning insulin. Other physician and participant characteristics were associated with beginning insulin at p ≤ 0.20 only in the four-regions-combined analysis. They were physician specialty (general practitioner/specialist) and sex, participant body mass index, regional location and presence of ≥1 macrovascular condition. High-density lipoprotein (HDL)-cholesterol, triglycerides and creatinine clearance were also eligible for four-regions-combined multivariable analysis but were not included because >20% of the data were missing. A multivariable backward logistic regression was then performed comparing premix insulin vs. basal insulin or other insulin vs. basal insulin. Using a backward procedure, all non-significant variables were removed one by one until all included variables reached a p-value of ≤0.05. To take into account differences between countries, country was included as a stratum in the multivariable analysis. Odds ratios (ORs) were calculated along with 95% CIs.

## Results

### Sites, Physicians and Participants

A total of 3061 participants were enrolled in the study. Thirty people were excluded from the analysis population (N = 3031) due to receiving insulin for >12 months before study entry (n = 23), having type 1 diabetes (n = 3) or diabetes due to pancreatitis (n = 2) or pregnancy (n = 1) and having no time between starting insulin and study entry (n = 1). The disposition and number of physicians (N = 314) and participants (N = 3061) by country and region are shown in Table 1. A substantial proportion of participants was contributed by all regions, except for the North American region represented by Canada alone (8.2%). The largest proportion of participants (35.3%) was from the countries designated as Southern Europe. One single country region, Japan, contributed a substantial proportion of the total study population (17.3%).

**Table 1:** Physician and participant disposition by country/region in people with type 2 diabetes starting insulin therapy

	Physicians*			
		
Region/country	Generalists (n)	Specialists (n)	Total, n (%)	Participants, N (%)
Canada†	6	17	23(7.3)	252(8.2)
Eastern Europe	0	49	50(15.9)	738(24.1)
Croatia			6(1.9)	53(1.7)
Russia			32(10.2)	536(17.5)
Ukraine			12(3.8)	149(4.9)
Japan†	1	62	63(20.1)	528(17.3)
Northern Europe	11	29	40(12.7)	461(15.1)
Finland			7(2.2)	125(4.1)
Germany			8(2.5)	87(2.8)
UK			25(8.0)	249(8.1)
Southern Europe	29	108	138(44.0)	1082(35.4)
France			88(28.0)	432(14.1)
Italy			22(7.0)	419(13.7)
Portugal			15(4.8)	166(5.4)
Spain			13(4.1)	65(2.1)
Total	47	265	314(100.0)	3061(100.0)

* Generalist/specialist not reported for two physicians.

† Canada and Japan are treated as single regions.

There were differences in baseline characteristics of the analysis population at the time of beginning insulin (Table 2). Mean age was 61 years, with diabetes duration from diagnosis of 11 years and body mass index of 29.3 kg/m^2^. Sex distribution showed male predominance, except for the Eastern European countries where women formed 75% of the sample. Ethnicity closely matched the country/region of origin—only Canada showed any significant diversity. Approximately 34% of the participants had a history of cardiovascular disease, lowest in Japan (25%) and highest in Eastern Europe (47%). The percentage of participants with microvascular disease was high overall (75%), being the least in Southern Europe (56%) and the greatest in Canada (86%). Approximately 15% were current smokers. Japanese participants had lower body weight and body mass index compared with the other regions. They also had a lower prevalence of diagnosed hypertension (48%).

**Table 2:** Baseline characteristics of the people with type 2 diabetes studied at the time of starting insulin

	Canada (n = 252)	Eastern Europe (n = 735)	Northern Europe (n = 460)	Southern Europe (n = 1073)	Japan (n = 511)	Total (N = 3031)
Age (years)	61 (11)	58 (8)	63 (11)	63 (11)	62 (10)	61 (10)
Sex (% female)	36	75	39	44	36	49
Ethnicity (%)						
Europid	81	94	92	44	0	59
Black	5	0	2	1	0	1
Asian	12	1	4	0	100	19
Other/missing	2	5	2	56	0	21
Body weight (kg)	92 (22)	83 (16)	90 (19)	80 (17)	62 (12)	80 (19)
Body mass index (kg/m^2^)	32.4 (7.6)	30.7 (5.4)	31.5 (6.3)	29.6 (5.9)	23.9 (4.0)	29.3 (6.3)
Duration of diabetes (year)	11 (7)	8(5)	9 (6)	12 (9)	12 (9)	11 (8)
History of microvascular disease (%)	86	93	79	56	80	75
History of macrovascular disease (%)	32	47	35	29	25	34
History of hypertension (%)	81	78	76	66	48	69
Physical activity, yes (%)	54.0	63.3	48.6	39.8	38.5	47.1
Current smoker (%)	14	8	18	15	25	15
HbA1c (%)	9.0 (1.8)	9.7 (1.9)	9.1 (2.0)	9.3 (1.9)	10.3 (2.0)	9.5 (2.0)
FPG (mmol/l)	10.9 (4.0)	11.7 (3.1)	10.7 (3.4)	11.8 (3.9)	11.9 (4.4)	11.6 (3.7)
PPG (mmol/l)	12.9 (4.5)	13.7 (3.6)	14.3 (5.3)	13.6 (4.4)	16.5 (5.3)	14.3 (4.6)
Total cholesterol (mmol/l)	4.4 (1.6)	6.0 (1.3)	4.6 (1.4)	5.0 (1.2)	5.2 (1.1)	5.2 (1.4)
LDL-cholesterol (mmol/l)	2.6 (0.9)	2.8 (1.2)	2.7 (0.9)	3.0 (0. 9)	3.15 (0.9)	2.9 (0.9)
HDL-cholesterol (mmol/l)						
Males	1.1 (0.3)	1.4 (0.5)	1.1 (0.3)	1.2 (0.4)	1.3 (0.4)	1.2 (0.4)
Females	1.2 (0.3)	1.6 (0.7)	1.4 (0.4)	1.3 (0.4)	1.5 (0.4)	1.4 (0.4)
Triglycerides (mmol/l)	2.1 (1.7)	2.8 (4.3)	2.5 (2.4)	1.9 (1.5)	1.8 (1.5)	2.1 (2.3)

FPG, fasting plasma glucose; HDL, high-density lipoprotein; LDL, low-density lipoprotein; PPG, post-prandial glucose.

Data are mean (s.d.) or % where indicated.

Participating physicians ranged in mean age from 44 years in Eastern Europe to 52 years in Japan. Overall type of physician practice was approximately equally distributed between office-based (33%), hospital-based (39%) and both office and hospital-based (28%) location. The majority of participating physicians (76%) were diabetes specialists (diabetologists/endocrinologists) who practised in an urban setting (75%). However, Japanese physicians were almost exclusively specialists (97%), their practices being a mixture of urban (46%) and rural (54%). In Eastern Europe, physicians were almost entirely female (92%) and were diabetes specialists (98%), practising in an urban setting (100%). Insulin was started almost exclusively under the supervision of diabetologists/endocrinologists in Eastern Europe (96%) as well as in Japan (94%), falling to 81% in Southern Europe, and still as high as two-thirds in Northern Europe (66%) and Canada (71%).

### Blood Glucose Control and Prior Glucose-Lowering Therapy

Overall, people starting insulin in the study had a mean HbA1c of 9.5% (80 mmol/mol) (Table 2). In Canada and Northern Europe, which had the lowest mean HbA1c prior to commencing insulin, >40% of participants nevertheless had baseline HbA1c ≥ 9.0% (75 mmol/mol). Japan had the highest mean HbA1c (10.3%, 89 mmol/mol) that was accounted for by higher post-prandial glucose (PPG) levels. Mean fasting plasma glucose (FPG) clustered around 11.6 mmol/l in the different regions and was lowest in Canada. PPG averaged 14.3 mmol/l. In general, blood glucose control was similar across the European regions.

Prior to beginning insulin, 7.0% of participants were receiving no recorded OGLDs, while 24.2, 48.7 and 20.1% received one, two or three or more OGLDs, respectively (Table 3). More Japanese participants (19.2%) and fewer Canadian (2.8%) participants had never received another glucose-lowering therapy, while almost half (48.8%) of the Canadian participants had received three or more OGLDs previously. By contrast, very few Eastern European participants had prior treatment with three or more OGLDs (3.0%). The most common OGLDs that had been prescribed for participants in the study at some time prior to starting insulin were sulphonylureas (received by 76.0% of participants), then biguanides (66.3%) and thiazolidinediones (21.2%). Fewer Japanese participants (29.0%) were using biguanides than in other regions, and Canada (87.3%), Northern Europe (78.9%) and Southern Europe (77.4%), all recorded higher usage. By contrast, prior use of *α*-glucosidase inhibitors (11.1%) was mostly by Japanese participants (34.6%). Overall, there were considerable similarities in treatment regimens between the European regions.

**Table 3:** Therapies before and at the time of starting insulin by country/region

	Canada (n = 252)	Eastern Europe (n = 735)	Northern Europe (n = 460)	Southern Europe (n = 1073)	Japan (n = 511)	Total (N = 3031)
Before insulin therapy						
Number of oral agents (%)						
None	2.8	5.2	4.3	4.5	19.2	7.0
Monotherapy	11.5	35.2	26.1	19.3	23.3	24.2
Dual therapy	36.9	56.6	49.1	53.3	33.3	48.7
Three or more	48.8	3.0	20.4	22.9	24.3	20.1
Types of oral agent (%)						
Biguanides	87.3	61.0	78.9	77.4	29.0	66.3
Sulphonylureas	83.7	86.4	71.1	72.2	69.9	76.0
Glinides	7.9	4.2	3.9	13.9	5.5	8.1
Thiazolidinediones	52.4	4.2	29.1	19.8	26.2	21.2
*α*-Glucosidase inhibitor	4.0	1.6	2.2	11.8	34.6	11.1
When starting insulin therapy						
Insulin (%)						
Basal insulin alone	53.6	59.7	61.5	62.8	6.3	51.6
Basal + mealtime insulin	8.7	16.6	7.2	12.4	26.2	14.6
Mealtime insulin alone	2.4	1.6	3.7	5.2	25.4	7.3
Premix insulin alone	35.3	21.5	27.6	12.9	36.8	23.1
Other	0	0.5	0	6.7	5.3	3.4
Oral agent number (%)						
None	23.4	25.6	22.0	28.0	51.3	30.0
Monotherapy	29.8	43.5	38.7	29.5	21.5	33.0
Dual therapy	33.7	29.9	34.6	34.1	17.8	30.4
Three or more	13.1	1.0	4.8	8.5	9.4	6.6
Oral agent type (%)						
Biguanides	68.7	43.1	66.3	56.4	21.3	49.8
Sulphonylureas	44.4	56.7	43.5	42.2	26.0	43.4
Glinides	4.0	2.9	2.0	10.4	2.0	5.3
Thiazolidinediones	17.5	3.3	8.9	6.9	15.7	8.7
*α*-Glucosidase inhibitor	2.0	0.3	0.9	6.4	20.9	6.2
Concomitant therapies (%)						
≥1 Concomitant therapies	90.5	85.9	92.2	81.8	66.7	82.5
Beta-blockers	31.7	27.5	41.3	23.6	9.2	25.5
Calcium channel blockers	24.6	15.0	25.4	20.0	29.7	21.6
Diuretics	35.3	36.2	37.4	28.1	10.4	29.1
ARIIB/ACE inhibitor	72.2	66.7	59.8	55.6	35.6	56.9
Statin	75.0	19.0	65.0	46.4	31.9	42.5
Antiplatelets	42.1	18.8	52.6	35.8	23.3	32.6
Anticoagulants	11.5	13.5	9.6	8.2	5.5	9.5

ARIIB/ACE, angiotensin II receptor blocker/ACE inhibitor.

### Choice of Insulin and Concomitant Therapies

Overall, 51.6% of the participants commenced insulin therapy with a basal insulin-only regimen, but this regimen was begun by only 6.3% of participants in Japan (Table 3). Premix insulin was commenced by 23.1%, with substantial variation between regions from 12.9% in Southern Europe to 35.3% in Canada and 36.8% in Japan. Other regimens were less frequently used with basal + mealtime 14.6% overall, but 26.2% in Japan, while mealtime insulin alone was commenced in 7.3% participants overall and in 25.4% in Japan.

When beginning insulin, 23.0% of participants discontinued oral agent therapy; the number includes the 7.0% not on such therapy previously. The reduction in the use of oral agents meant that the proportion using a single oral agent in combination with insulin monotherapy increased markedly, except in Japan, overall to 33% of participants. The proportion on dual oral agent therapy with insulin fell in the European regions, where it was high prior to beginning insulin, and in Japan, but in Canada was maintained (33.7%) as the higher prior proportion on triple therapy dropped one agent (Table 3).

In Canada and Japan, a substantial proportion (17.5 and 15.7%) of people continued to take thiazolidinediones once started on insulin (Table 3). Overall, the percentage of people continuing metformin or sulphonylurea (often in combination) was not dissimilar, though with higher metformin use in Canada, and Northern Europe, and to some extent Southern Europe.

Eighty percent of participants overall were taking at least one other medication of any kind, although only two of three were in Japan (Table 3). In keeping with a lower incidence of hypertension, fewer participants in Japan were taking *β*-adrenergic blockers, diuretics and angiotensin-2 blockers or angiotensin-converting enzyme inhibitors than in other regions. Statins were used less in Eastern Europe (19%) than in other regions.

### Associations With Different Types of Insulin

The association between baseline HbA1c and glucose-lowering therapies with different insulin regimens when beginning insulin was analysed separately for Japanese participants, as their baseline characteristics differed from those of the other regions (Table 4). Most insulin regimens were begun in people with mean baseline HbA1c ≥8.8% (72 mmol/mol), except for mealtime insulin, where participants had a mean HbA1c of 8.2% (66 mmol/mol). Overall, >60% began with basal insulin, mostly together with dual oral agent therapy (43%), taking biguanides (64.9%) and sulphonylureas (63.3%), while fewer beginning basal insulin had no concomitant OGLDs (11.2%) than with other regimens.

**Table 4:** Blood glucose control and oral glucose-lowering medications at the time of starting insulin

	Basal insulin alone	Basal + mealtime insulin	Mealtime insulin alone	Premix insulin	Other
Number (non-Japan/Japan)	1531/32	310/134	91/130	512/188	76/27
Baseline HbA1c (% units, mean (s.d.) [95% CI]					
Excluding Japan	9.2 (1.8) [9.1, 9.3]	9.8 (2.1) [9.5, 10.0]	8.2 (1.6) [7.9, 8.6]	9.8 (2.0) [9.6, 9.9]	8.8 (1.7) [8.3, 9.7]
Japan	9.8 (1.5) [9.2, 10.3]	10.8 (2.4) [10.4, 11.2]	10.1 (1.9) [9.8, 10.5]	10.1 (1.8) [9.8, 10.4]	10.0 (2.4) [9.1, 11.0]
Number of treatments (%, 95% CI)					
Excluding Japan					
None	11.2 [9.6, 12.9]	60.0 [54.3, 65.5]	44.0 [33.6, 54.8]	38.9 [34.6, 43.2]	68.4 [56.7, 78.6]
Monotherapy	37.6 [35.1, 40.0]	27.1 [22.2, 32.4]	24.2 [15.8, 34.3]	38.1 [33.9, 42.4]	17.1 [9.4, 27.5]
Dual therapy	43.0 [40.5, 45.6]	12.3 [8.8, 16.4]	29.7 [20.5, 40.2]	18.9 [15.6, 22.6]	11.8 [5.6, 21.3]
≥3 Oral therapies	8.2 [6.9, 9.7]	0.6 [0.1, 2.3]	2.2 [0.3, 7.7]	4.1 [2.6, 6.2]	2.6 [0.3, 9.2]
Japan					
None	9.4 [2.0, 25.0]	72.4 [64.0, 79.8]	62.3 [53.4, 70.7]	34.6 [27.8, 41.8]	59.3 [38.8, 77.6]
Monotherapy	28.1 [13.7, 46.7]	20.1 [13.7, 27.9]	15.4 [9.7, 22.8]	25.0 [19.0, 31.8]	25.9 [11.1, 46.3]
Dual therapy	34.4 [18.6, 53.2]	6.7 [3.1, 12.4]	17.7 [11.6, 25.4]	23.4 [17.6, 30.1]	14.8 [4.2, 33.7]
≥3 Oral therapies	28.1 [13.7, 46.7]	0.0 [0.0, 10.6]	4.6 [1.7, 9.8]	17.0 [11.9, 23.2]	0.0 [0.0, 12.8]
Type of oral therapy (%) [95% CI]					
Excluding Japan					
Biguanides	64.9 [62.5, 67.3]	33.2 [27.9, 38.9]	49.4 [38.7, 60.2]	48.8 [44.4, 53.3]	27.6 [18.0, 39.1]
Sulphonylureas	63.3 [60.9, 65.8]	17.3 [13.2, 22.0]	23.6 [15.2, 33.8]	26.7 [22.9, 30.8]	13.2 [6.5, 22.9]
Glinides	7.6 [6.3, 9.1]	1.7 [0.5, 3.8]	9.0 [4.0, 16.9]	4.3 [2.7, 6.5]	1.3 [0.0, 7.1]
Thiazolidinediones	9.1 [7.7, 10.7]	1.7 [0.5, 3.8]	5.6 [1.8, 12.6]	6.7 [4.7, 9.3]	0.0 [0.0, 4.7]
*α*-Glucosidase inhibitor	3.7 [2.8, 4.8]	1.3 [0.4, 3.4]	3.4 [0.7, 9.5]	2.2 [1.1, 3.9]	6.6 [2.2, 14.7]
Japan					
Biguanides	34.4 [18.6, 53.2]	15.3 [9.3, 23.0]	19.3 [12.7, 27.6]	29.0 [22.5, 36.1]	15.4 [4.4, 34.9]
Sulphonylureas	56.3 [37.7, 73.6]	4.2 [1.4, 9.6]	23.5 [16.2, 32.2]	43.7 [36.4, 51.2]	7.7 [0.9, 25.1]
Glinides	9.4 [2.0, 25.0]	0.0 [0.0, 3.1]	1.7 [0.2, 5.9]	2.7 [0.9, 6.3]	0.0 [0.0, 13.2]
Thiazolidinediones	28.1 [13.7, 46.7]	8.5 [4.1, 15.0]	9.2 [4.7, 15.9]	23.5 [17.6, 30.3]	26.9 [11.6, 47.8]
*α*-Glucosidase inhibitor	53.1 [34.7, 70.9]	12.7 [7.3, 20.1]	16.8 [10.6, 24.8]	29.0 [22.5, 36.1]	7.7 [0.9, 25.1]

CI, confidence interval.

In Japan, there was no difference in baseline HbA1c for participants starting on any insulin regimen (Table 4). Fewer participants beginning basal insulin had no concomitant glucose-lowering medications (9.4%) than with other insulin regimens. Otherwise, there were no differences between regimens in the number and types of oral glucose-lowering medications.

### Factors Influencing Choice of Initial Insulin Regimen

**Figure 1:**
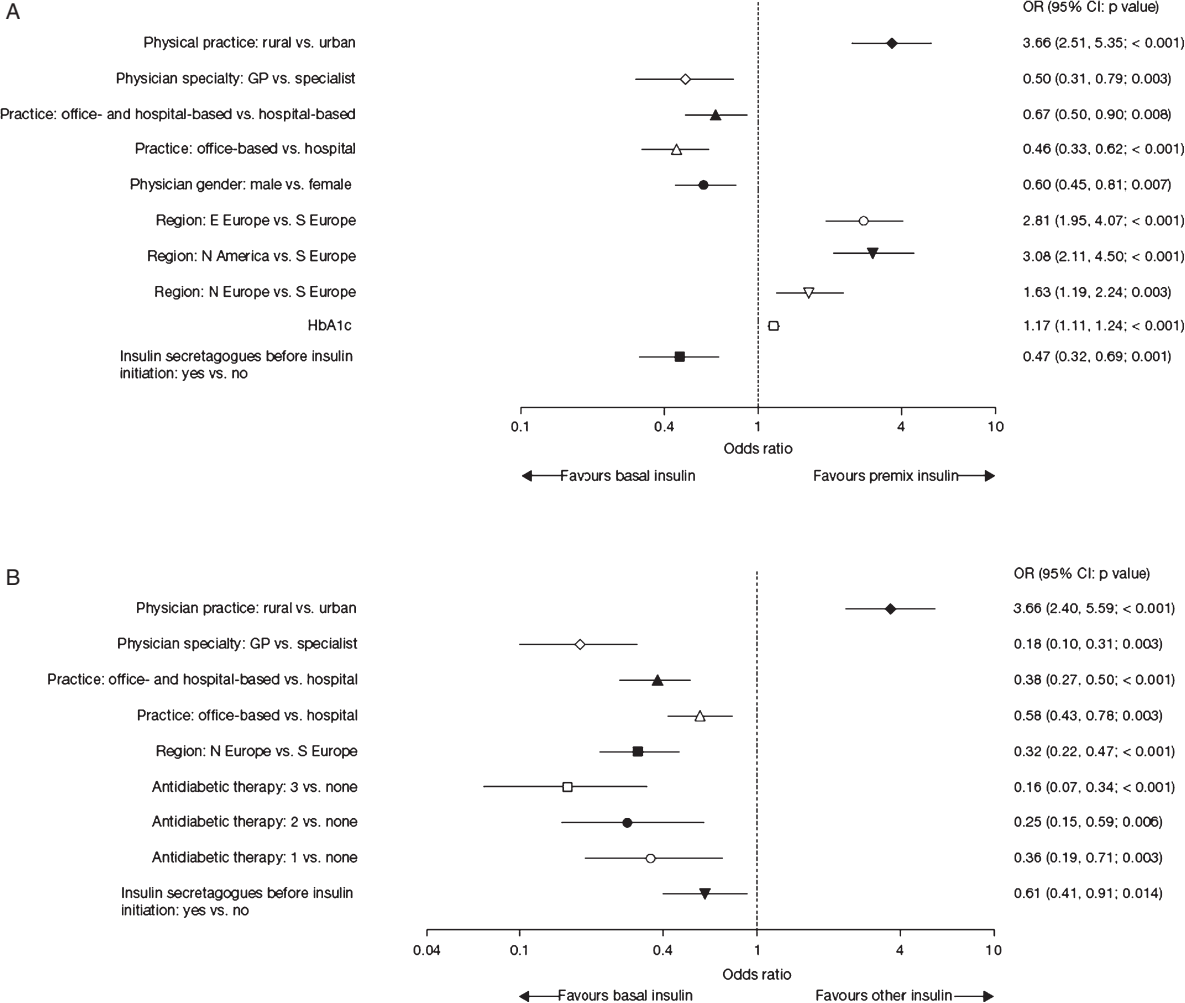
Factors influencing choice of initial insulin therapy in people with type 2 diabetes, as determined by multivariable analysis of premix vs. basal insulin (A) and other insulin treatments vs. basal insulin (B) in all regions except Japan. The model was based on 2200 observations in A and 469 observations in B. There was no evidence of over-dispersion as the ratio of deviance to degrees of freedom was 0.86. HbA1c odds are per 1.0% units increase. For each pair of factors (e.g. rural vs. urban), odds ratios (ORs) > 1 indicate that the first of the pair (e.g. rural) favours premix insulin over basal insulin (A) or other insulin over basal insulin (B); ORs < 1 indicate that the first of the pair (e.g. general practitioner) favours basal insulin.

Factors influencing the choice of premix insulin compared with basal insulin alone, in the four-regions-combined (excluding Japan), were physician specialty, practice location and type and sex of physician, and participant region, baseline HbA1c and prior insulin secretagogue therapy (figure 1A). Preference for basal insulin was associated with physicians who were general practitioners vs. specialists, physicians practising in an urban vs. rural location, those with an office-based practice and male physicians; for participants, preference for basal insulin was associated with less elevated baseline HbA1c levels, prior secretagogue therapy and living in the South Europe region. Factors influencing the use of basal insulin regimen compared with other insulin regimens (excluding premix insulin) were physician specialty, geographical location and practice type, and with participant region, prior oral agent therapy and use of insulin secretagogue therapy (figure 1B). Preference for basal insulin was associated with physicians who were general practitioners, those practising in an urban location and those whose practice was office based and with participant prior oral agent therapy and secretagogue therapy and with participants living in Southern Europe compared to Northern Europe.

The only factor influencing the choice of insulin regimen in Japan was geographical location of the physician. When comparing premix insulin with basal insulin, preference for starting with basal insulin was associated with physicians who were practising in an urban location [OR rural vs. urban 0.42; (95% CI: 0.18, 0.99); p = 0.047]. The same was true when comparing other insulin regimens with basal insulin (OR rural vs. urban 0.32; 95% CI: 0.14, 0.74; p = 0.008), while there was also weak evidence that older physicians may prefer to begin with basal insulin (OR 0.71; 95% CI: 0.49, 1.02; p = 0.06).

## Discussion

Insulin therapy is not usually started when T2DM is diagnosed, except where co-morbid conditions or therapy contribute to more extreme hyperglycaemia. Only when the target HbA1c level is no longer attained with one or more oral glucose-lowering agents are insulin injections, or sometimes glucagon-like peptide-1 mimetics, normally started. Participants in the CREDIT study in general followed that pattern as more than two-thirds had previously taken two or more oral agents, mostly metformin and sulphonylureas. However, in general, they had been allowed to deteriorate to levels of glucose control well over target levels, as evidenced by elevated HbA1c, FPG and PPG. Similar poor glycaemic control across countries in people with T2DM starting insulin has been reported previously [9,10] and appears to be common in clinical practice worldwide [11].

Duration of diabetes when starting insulin was generally around 10 years, but it is not possible from the current study to know how long uncontrolled hyperglycaemia had been allowed to persist before starting insulin, nor what other therapeutic attempts had been made to control it. The published literature suggests that HbA1c is higher at each step or addition of a new medication [12], suggesting that the late starting of insulin is just another part of a worsening continuum of failure to achieve glucose control targets. This in turn suggests that either glucose control deteriorates faster than physicians believe that they need to titrate therapy or that the barriers to therapy titration worsen with number of therapies or both.

That the problem is a systematic management issue related to use of therapies and control of metabolic risk factors, rather than control of glucose levels and use of insulin, is supported by the observation that other management was also not optimal. While the problem of obesity may be unmanageable in many people with current therapeutic tools, the elevated mean low-density lipoprotein (LDL)-cholesterol and triglyceride levels and omissions of therapy to control these, in addition to the similar blood pressure management findings, suggest a global failure of preventative care rather than a problem related to one specific cardiovascular risk factor. In this regard, it can be seen that statin use was lowest in Eastern Europe and Japan, the countries with the highest LDL-cholesterol levels at the time of insulin initiation. This prescribing pattern was echoed for antiplatelet agents, but for blood pressure lowering agents, the pattern is less clear, partly due to different patterns of use of different antihypertensive agents (such as high renin angiotensin system blockade in Eastern Europe) and the lower prevalence of hypertension in the Japanese participants.

Insulin therapy was begun at a similar age on average across the regions studied, but other characteristics were strikingly diverse. While there may be many reasons for the diversity, they almost certainly include differences across the regions/countries in pharmaceutical company marketing campaigns and the availability of diabetes therapies and of nurses to both educate patients and influence the choice of treatment. The reason for the high proportion of women in the Eastern European region could be that men are more reluctant to visit doctors and health issues awareness and the level of self-care is much lower in men in these societies. Easier to explain is the relatively light and thin Japanese cohort, though disappointingly they also have the highest HbA1c levels, which are explained by high PPG levels rather than FPG. It has long been suggested that T2DM in Japan is characterized more by insulin deficiency than insulin insensitivity relative to ‘Western’ T2DM [13] and our data, and the lower rates of vascular disease, would be consistent with this. A similar diversity across some of the same countries/regions was observed in people with T2DM initiating biphasic insulin aspart 30 [10], including a high percentage of women in Russia. Because such a pattern of hyperglycaemia might be expected to determine different approaches to oral agent and insulin therapy, we disaggregated Japanese data before further analysis of the determinants of the type of insulin therapy.

Perceptions of care may also be influencing the use of other therapies up to the time insulin was started. Thus, the relative over-use of sulphonylureas rather than metformin in Japan might be a reflection of the lower body mass index and the belief that insulin deficiency was the primary problem [14]. Although thiazolidinedione use was even lower in Eastern Europe, a factor here would be high cost and lack of reimbursement and/or unavailability of these proprietary drugs, while in Canada, with the most obese population of the regions studied, the largest proportion of people were managed with a combination of metformin, a sulphonylurea and an insulin sensitizer before consideration of insulin therapy. In general on starting insulin, any use of thiazolidinediones was markedly reduced where used in a significant proportion of the population, reflecting their limited indications in this combination, while in general, metformin was continued. However, in 24.9% of people taking it, metformin was stopped when starting insulin, perhaps a surprise as it is known to be weight-, dose- and hypoglycaemia sparing. Part of the explanation here may be people stopping metformin because of developing contraindications, with insulin being started as a substitute glucose-lowering medication.

When determining what influenced the choice of insulin regimen the physician factors of specialty type, specialty location, age and sex may be related to the mix of people with diabetes under their care, and the traditions of insulin therapy at the time of training, together with acquired expertise in insulin dose adjustment. This is supported by the observation that basal insulin, often judged easier to use and with less risk of hypoglycaemia, is seemingly preferred by general practitioners, in an office-based environment, and in urban locations, while premix is more widely used in specialist care. General practitioner preference for basal insulin may be related to them placing greater importance on patient adherence when initiating insulin than do specialists [8], with patients more likely to adhere to a single rather than multiple daily injections. Less easy to explain are regional differences, with Canada and Eastern Europe preferring premix insulin over basal insulin when compared to Southern Europe. One possible explanation here, which is difficult to address further, is that this is a consequence of previous practice and local marketing practices. On the other hand, use of oral agents, and in particular, insulin secretagogues is associated with the use of basal insulin, perhaps a more natural partner for these agents than with premix insulin or other insulin regimens. By contrast, HbA1c level had little influence on the choice of insulin, despite some suggestion that basal insulin might be more suitable at better levels of blood glucose control, and premix where it had deteriorated further, and despite both general practitioners and specialists citing the extent of HbA1c elevation as the major consideration for starting insulin [8].

Our analysis has some limitations. In particular, while attempts were made to reduce bias in choice of centres taking part in the study, it remains possible that centres more likely to take part might have good relationships with the sponsor, a suggestion somewhat countered by the frequent use of premix insulin in the population studied. Centres willing to contribute data may have different standards of practice from the bulk of insulin prescribers, another unknown or latent factor. Further, these biases could be operating differently between the global regions studied or even between practitioners of different specialties or working in different environments. While observational studies cannot provide explanations for the relationships observed, they do provide information that is complementary to that of randomized clinical trials [15].

We conclude nevertheless that the apparent glucose control levels when starting insulin are poor in all studied regions and that this seems to reflect a general ‘failure’ of preventative care. The choice of insulin also varies by region and by the type and situation of the practising physician for reasons that appear to have no relationship to the metabolic state of the patient. The exception to this is the Japanese population, where different demographic and pathophysiological characteristics may partly explain divergent choices of therapy.
